# Distinct correlations between clinical and laboratory-based instrumented balance measures in individuals with multiple sclerosis: a cross-sectional study

**DOI:** 10.1038/s41598-026-58829-9

**Published:** 2026-06-23

**Authors:** Melanie Bommer, Jonas Freiermuth, Laurin Bachmann, Mara Augstburger, Corina Schuster-Amft, Zorica Suica, Hans Ulrich Gerth, Leo H. Bonati, Katrin Parmar, Frank Behrendt

**Affiliations:** 1 Research Department, Reha Rheinfelden, Rheinfelden, Switzerland; 2 Swiss Paraplegic Research, Nottwil, Switzerland; 3 Department of Health Sciences and Technology, ETH Zurich, Zurich, Switzerland; 4 Department for Sport, Exercise and Health, University of Basel, Basel, Switzerland; 5 School of Engineering and Computer Science, Bern University of Applied Sciences, Burgdorf, Switzerland; 6 Department of Medicine, University Hospital Münster, Münster, Germany; 7 Department of Clinical Research, University of Basel, Basel, Switzerland; 8 Department of Neurology, University Hospital Basel, Basel, Switzerland; 9 Translational Imaging in Neurology (ThINk) Basel, Departments of Head, Spine and Neuromedicine and Biomedical Engineering, University Hospital Basel and University of Basel, Basel, Switzerland

**Keywords:** Health care, Medical research, Neurology, Neuroscience

## Abstract

Multiple sclerosis (MS) frequently leads to balance impairments and an increased risk of falls. Because laboratory-derived stability metrics are rarely feasible in clinical routine, clinicians commonly rely on functional balance assessments. This exploratory cross-sectional study examined associations between six clinical balance tests (DGI, BBS, TUG, Mini-BESTest, SSST, CTSIB) and laboratory-derived measures of static and dynamic balance, including margin of stability (MoS), whole-body angular momentum (WBAM), and center of pressure (CoP) in 30 people with MS (EDSS 3.6 ± 1.1). Patients completed 3D gait analysis, force-plate posturography, and clinical tests. Spearman correlations and nonparametric bootstrapping were used to estimate associations and 95% confidence intervals. Clinical test scores generally showed moderate-to-strong correlations with CoP measures, with the strongest association observed for the BBS (*r* = 0.76). For dynamic outcomes, larger MoS values were associated with poorer clinical test performance, most prominently for the TUG, suggesting that MoS may partly reflect compensatory gait strategies rather than superior balance capacity. Frontal-plane WBAM showed its strongest correlation with the TUG (*r* = 0.67). In conclusion, the BBS was most closely associated with static balance, whereas the TUG showed the most consistent associations across selected static and dynamic laboratory-derived balance measures.

**Supplementary Information:**

The online version contains supplementary material available at https://doi.org/10.1038/s41598-026-58829-9.

## Introduction

Balance control is frequently compromised in people with MS (pwMS), reflecting the combined impact of sensory, motor, and cognitive impairments; postural instability is reported in the vast majority of individuals over the disease course^1^. Falls are a major clinical consequence: a position paper and narrative review summarizing international data indicates that the incidence of falls in pwMS exceeds 50%, with substantial proportions of individuals experiencing recurrent and injurious events^2^. Consistent with this, a recent systematic review and meta-analysis reported that, within a one-year period, 42.5% of pwMS experienced one fall and 44.7% reported repeated falls^3^. Beyond immediate physical consequences, falls contribute to fear of falling, activity curtailment, and downstream deconditioning, thereby amplifying disability and participation restrictions^2^. Importantly, perceived risk (e.g., balance confidence or self-efficacy) can diverge from physiological fall risk in MS, underscoring the need for reliable balance assessment^4,5^.

Clinically, balance is commonly treated as a task-specific construct and differentiated into static balance, maintaining postural stability over a fixed base of support, and dynamic balance maintaining stability while the center of mass is in motion^6,7^. This conceptual distinction is reflected in the variety of routine clinical assessments. In pwMS, balance dysfunction has been conceptualized as a combination of decreased ability to maintain position (static component), reduced ability to move toward limits of stability, and delayed responses to postural perturbations (dynamic components)^8^. Because falls in pwMS occur most frequently during dynamic activities, dynamic balance testing adds relevant information beyond quiet-stance metrics alone^9^.

Balance performance can be assessed using a spectrum of approaches, ranging from straightforward clinical instruments such as the Berg Balance Scale^10^ and the Dynamic Gait Index^11^ to more elaborate laboratory-derived metrics, including the MoS^12^, WBAM^13^ or force-plate posturography^14^. Hof et al. introduced the principle of the MoS which describes the distance of the vertically extrapolated center of mass (xCoM) to the base of support (BoS). In this way, dynamic balance can be assessed in anterior-posterior and medio-lateral directions using MoS, a well-established metric. Within the extrapolated center-of-mass framework, a positive MoS is generally interpreted as mechanical stability, whereas a negative MoS indicates that corrective action is required to avoid loss of balance. However, the clinical interpretation of larger positive MoS values can be complex, particularly when individuals adopt compensatory gait strategies^12,15,16^.

WBAM represents the total momentum generated around the body’s CoM by all segments of the body. During normal, stable walking, this value should be small, since the contributions of different segments cancel each other out^13,17^. However, in subjects with poorer balance control and higher likelihood of falling, it has been shown, that the control of WBAM decreases and therefore the values reach higher peaks^18–23^. Although all three WBAM components (sagittal, frontal and transverse) are informative, the frontal-plane (mediolateral) component is often particularly clinically relevant. Increased frontal-plane WBAM peaks may reflect reduced mediolateral balance control and greater reliance on compensatory recovery strategies^13^.

In this study, also static postural sway was evaluated. Static balance can be quantified using force-plate posturography by recording the center of pressure (CoP) trajectory during quiet standing, where CoP fluctuations reflect ongoing neuromuscular adjustments of the postural control system^24^. CoP-based summary metrics are widely used to characterize postural steadiness, including spatial measures of sway such as sway area^25^.

Vistamehr et al. investigated whether commonly used clinical balance assessments align with laboratory-derived measures of mediolateral dynamic stability in individuals with post-stroke hemiparesis^18^. BBS and DGI scores were related to treadmill-based estimates of MoS and the WBAM range. The authors reported moderate correlations among the measures. The pattern found for post-stroke hemiparesis likely does not directly translate to people with MS. In MS, balance impairment is typically multifactorial and consists of combinations of weakness/spasticity, sensory–proprioceptive deficits, incoordination, visual and cognitive contributions, and fatigue^26^. Instrumented dynamic balance measures (including MoS-based outcomes and related variability metrics) have been linked to physiological impairments and fall status in MS^27^, supporting the rationale to specifically test clinical–laboratory associations also in this population.

Since the complex measures of MoS, WBAM and static posturography using force plates are usually derived from 3D-motion analysis and the equipment and time needed for this procedure is not always available, clinicians usually rely on a range of functional assessments. In clinical practice, a range of additional assessments is commonly used. These include for instance, in addition to the BBS and the DGI, the Mini-Balance Evaluation Systems Test (Mini-BESTest)^28^, the Timed Up and Go (TUG) test^29^, the Six Spot Step Test (SSST)^30^, and the Clinical Test of Sensory Interaction on Balance (CTSIB)^31^. The Mini-BESTest was developed to capture multiple domains of primarily dynamic postural control within a brief clinical format, whereas the TUG operationalizes functional mobility as a time-based performance metric that integrates transfers, turning, and walking. The SSST emphasizes lower-limb coordination and dynamic balance during rapid stepping, while the CTSIB probes sensory contributions to postural stability by systematically manipulating visual and somatosensory conditions during stance, thereby increasing reliance on vestibular input.

The purpose of this study was to assess static and dynamic balance using the BBS, DGI, Mini-BESTest, TUG, SSST, and CTSIB, alongside laboratory-derived measures of MoS, WBAM and static postural sway to compare the clinical test scores with the lab-based results. To examine these associations, we aimed to include a different patient population distinct from that studied by Vistamehr et al.^18^, specifically pwMS.

## Methods

### Patients and study design

In this cross-sectional study, pwMS were included if they were medically stable and had an Expanded Disability Status Scale (EDSS) score between 1.0 and 6.0. PwMS were excluded if they had experienced a relapse or had undergone systemic cortisone therapy within the last 30 days, or if they had any additional neurological disease. The study protocol was approved by the Ethikkommission Nordwest- und Zentralschweiz (EKNZ), Basel, Switzerland (reference number 2023 − 01935) and conformed to the Declaration of Helsinki. All patients provided written informed consent before the start of the data collection.

### Outcome measures and test procedure

BBS, DGI, Mini-BESTest, TUG, SSST, and the CTSIB data were collected for each participant according to standard procedures. Within less than a week, 3D data were collected at 100 Hz using an 8-camera motion capture system (Vicon Motion Systems, Oxford, UK) while participants walked without assistance at their self-selected walking speed during multiple trials. For each participant, full-body kinematics from 20 gait cycles of each side were analyzed to compute the MoS and the WBAM. Static postural sway was evaluated by analyzing the 95% ellipse area of the CoP path^32^, a commonly reported variable in posturography. As CoP reliability depends on sampling duration and repeated trials, we acquired two 60-s quiet-standing recordings.

### Sample size estimation and statistical analyses

The required sample size was determined a priori using Fisher’s *z* transformation of Pearson’s correlation. Assuming a minimally relevant, clinically meaningful correlation of |ρ| = 0.50 with 80% power at a two-sided α = 0.05, *n* = 30 participants are required.

With regard to the WBAM analysis, a 15-segment body model with corresponding segment-mass proportion was used^17^ with the whole-body CoM (center of mass) calculated as the weighted average of each segment CoM. For each segment, the WBAM was calculated as the sum of each body segment momenta with respect to the CoM in the frontal, sagittal, transversal plane^13,17^. The WBAM was quantified on a step-by-step basis and normalized by the product of the body mass, CoM height, and walking velocity^13,17^. For each step, the absolute peak magnitude was extracted as the indicator of rotational imbalance^13^.

The anterior MoS was defined as the distance, measured in the forward walking direction, between the extrapolated center of mass (xCoM) and the marker placed on the ipsilateral second metatarsal head. The lateral MoS was defined as the distance in the mediolateral direction between the xCoM and lateral malleolus. Both MoS measures were evaluated at heel strike; positive values indicated mechanical stability, meaning that the xCoM lay posterior and medial to the corresponding foot markers for the anterior and lateral MoS, respectively^33^.

Non-parametric Spearman’s correlation analyses were performed between the clinical balance scores and the laboratory-based measures. We additionally applied a non-parametric bootstrap procedure^34^(1,000 resamples) to each pairwise association to obtain empirical 95% confidence intervals for the correlation estimates.

## Results

**Fig. 1:**
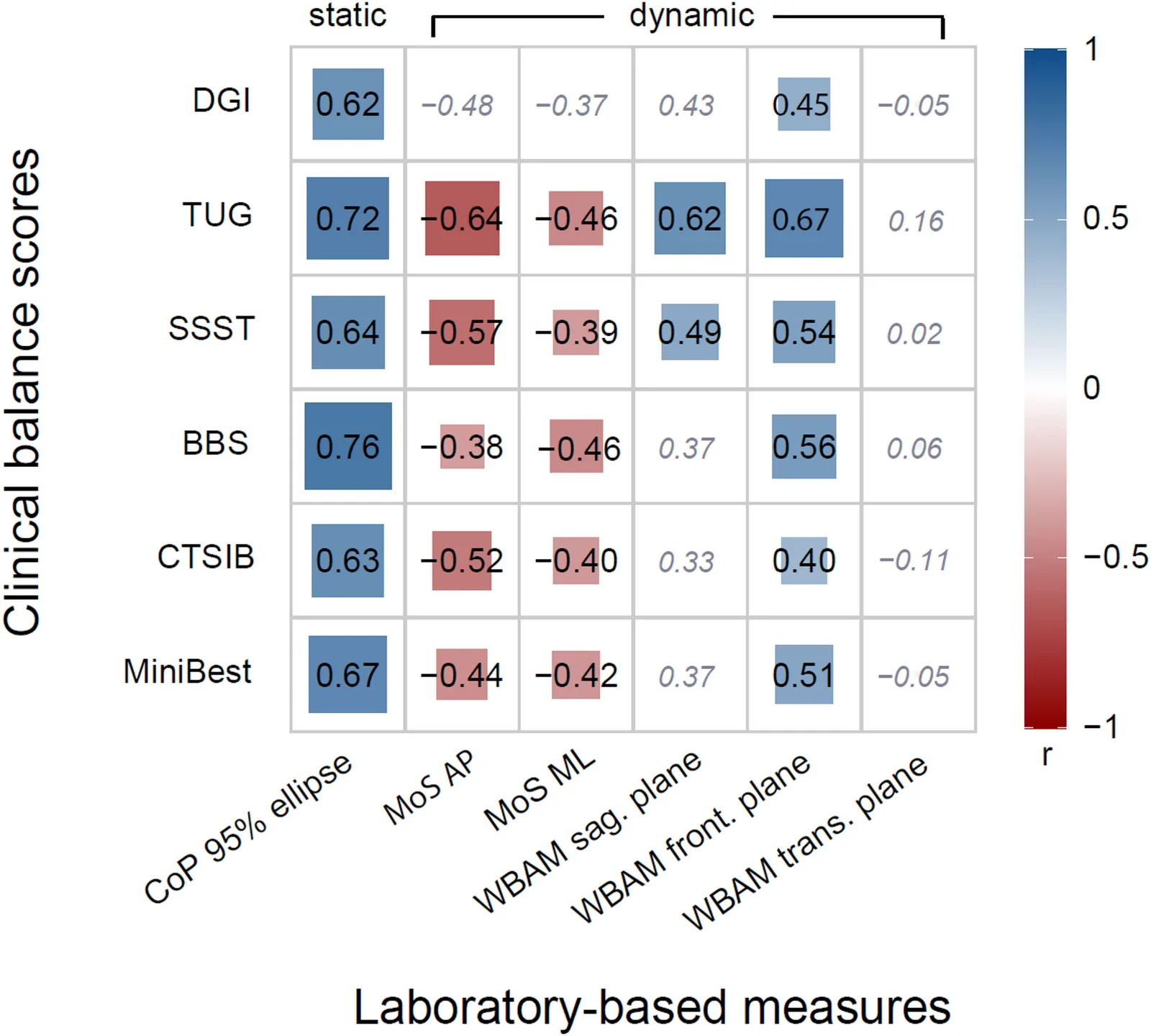
Correlation matrix for pairwise comparisons. The r-values are color-coded (see scale), and the size of the background squares is scaled according to magnitude and direction. Associations are greyed out and displayed in italics if the bootstrap confidence interval included *r* = 0 or did not include |r| ≥ 0.5. BBS = Berg Balance Scale; CoP = Center of Pressure; CTSIB = Clinical Test of Sensory Interaction on Balance; DGI = Dynamic Gait Index; MoS = Margin of Stability; SSST = Six Spot Step Test; TUG = Timed Up and Go Test; WBAM = Whole-Body Angular Momentum; AP = anteroposterior; ML = mediolateral.

Thirty outpatients with MS (age: 49.6 ± 10.5 years; 21 female) with a mean Expanded Disability Status Scale (EDSS) score of 3.6 ± 1.1 were enrolled in the study (please see Supplementary Table 1 for patient characteristics). Most participants were diagnosed with relapsing-remitting MS; only three had primary progressive MS and two had secondary progressive MS. For an overview of the individual correlation coefficients from the pairwise comparisons, please refer to Fig. 1. Please note, for some scores, the sign was reversed a priori to align lower versus higher scores with poorer versus better balance performance, respectively, to ensure a consistent directionality across all measures.

Clinical assessment scores showed moderate-to-strong positive associations with the static balance measure CoP 95% ellipse area, with the strongest association observed for the BBS (*r* = 0.76). Conversely regarding the dynamic balance measures, all clinical tests exhibited moderate to strong negative correlations with the MoS in both the anterior–posterior and medio-lateral directions, with the highest coefficients observed for the TUG (*r* = -0.64 and − 0.46, respectively). With respect to WBAM, correlations ranged from weak to strong in both the frontal and sagittal planes, with higher coefficients detected for the frontal plane (with TUG *r* = 0.62). In contrast, no consistent associations were found between transverse-plane WBAM and the clinical test scores.

In Fig. 1, associations were visually de-emphasized if the bootstrap confidence interval included *r* = 0 or did not include a correlation of at least moderate magnitude (|r| = 0.5).

For an overview of all pairwise comparisons across all assessed parameters, please see the Supplementary Fig. 1 including correlation coefficients, confidence intervals of the bootstrap analysis and significance values. It provides an overview of all comparisons, including the EDSS score, number of falls, clinical test scores, and laboratory-based measures.

## Discussion

This study examined concordance between commonly used clinical balance assessments (DGI, BBS, TUG, Mini-BESTest, SSST, CTSIB) and motion-analysis–derived measures reflecting static quiet and dynamic balance control (CoP, WBAM, MoS). We observed the strongest positive associations between the more static force-plate-based sway and the BBS, which is a clinical test with a pronounced standing component. This is plausible given that the BBS includes several standing and quasi-static balance tasks that may overlap with the demands of quiet-stance posturography^35^.

In contrast, all associations of the clinical tests with MoS were negative in both the anteroposterior and mediolateral directions after harmonizing scale direction via sign conventions. While MoS is commonly “mechanically” interpreted as a parameter of dynamic stability derived from the extrapolated CoM framework^36^, its clinical interpretation can be non-trivial because individuals may adopt conservative gait strategies, such as taking wider steps and other step-parameter adaptations that increase aspects of MoS without necessarily indicating better underlying balance capacity^33,37^. Thus, in populations with gait or balance deficits, larger MoS values may partially reflect compensation to achieve stability rather than superior balance control per se, implying that MoS may index balance impairment only indirectly when compensatory strategies dominate^33,38^.

To address this ambiguity, we additionally considered WBAM, which provides a mechanics-based measure that integrates segmental motions and inertia and has been proposed as a sensitive descriptor of dynamic balance control during walking^39^. MoS can provide insight into foot placement mechanisms, whereas angular-momentum analysis provides information on both foot placement and the generation of appropriate ground-reaction forces to maintain dynamic balance^18^. We found positive associations for WBAM in the frontal and sagittal planes, whereas transverse-plane WBAM showed no consistent relationships with the clinical tests.

Interestingly, the TUG, which incorporates also walking and turning, showed the most consistent associations with the ‘dynamic’ laboratory outcomes examined here. A plausible explanation is task specificity: the TUG is a time-based composite mobility task (sit-to-stand, straight walking, a 180° turn, return walking, stand-to-sit), so its score is strongly driven by spatiotemporal gait performance and turning control, which are the same domains that largely determine laboratory “dynamic” outcomes^29^. In instrumented analyses, step length and cadence explain most variance in the straight-walking TUG subtasks, and single-support timing contributes importantly to the turning subtask, underscoring why TUG can align especially well with mechanics-derived gait stability metrics^40^. As impaired individuals may adopt a conservative gait strategy that can increase MoS without necessarily reflecting better balance control, it would also simultaneously worsen TUG performance via slower gait and turning^41^. The positive association with both frontal and sagittal WBAM is plausible because turning and transitions require generating and then arresting WBAM, and turn initiation and termination involves angular-momentum regulation across these two planes^42^.

In pwMS, interpreting the results requires accounting for disease-specific mechanisms that may differ from patient populations. MS-related balance impairment is typically multifactorial and heterogeneous, with prominent contributions from slowed somatosensory conduction and impaired central sensory integration, which manifest as increased quiet-stance sway and exaggerated instability when sensory conditions are manipulated, which may partly explain why standing-oriented assessments such as the CTSIB and static components of the BBS showed associations with force-plate CoP sway metrics in the present cohort^8^. For dynamic balance, MS is also characterized by frequent adoption of altered locomotor strategies that may increase mechanical stability indices without necessarily indicating better underlying balance capacity, but possibly reflecting compensation as much as impairment^33^. The additional use of WBAM is therefore particularly relevant also in MS, given evidence of altered rotational control during functional tasks such as gait initiation in MS^43^. TUG includes tasks which are especially sensitive to balance confidence and mobility limitations in MS and may amplify the expression of both impairment and compensatory control strategies captured by MoS and frontal-/sagittal-plane WBAM^44^.

### Limitations

Several limitations should be considered when interpreting the findings of this study. First, the sample size was modest and was powered to detect correlations of at least moderate magnitude. Consequently, the present analyses should be regarded as exploratory, and the observed correlation pattern requires confirmation in larger independent cohorts. Relatedly, multiple pairwise associations were examined across several clinical and laboratory-derived outcomes. Although bootstrap confidence intervals were used to characterize the precision and robustness of the estimates, no formal correction for multiplicity was applied; therefore, individual associations should not be overinterpreted in isolation.

Second, the cross-sectional design precludes conclusions about causality or predictive validity. The study assessed associations between clinical tests and biomechanical measures at one time point, but it cannot determine whether a given clinical test predicts future falls, disease progression, or responsiveness to rehabilitation. Future studies should incorporate prospective fall monitoring and longitudinal follow-up to evaluate whether the identified clinical–biomechanical associations translate into clinically meaningful outcomes.

Third, the interpretation of dynamic stability metrics is inherently influenced by gait strategy. MoS is affected by spatiotemporal parameters such as step width, step length, walking speed, and foot placement. In individuals with impaired balance, larger MoS values may therefore reflect compensatory strategies rather than superior dynamic stability. Although this interpretation is central to the present findings, the study was not designed to disentangle impairment-related instability from adaptive compensation.

Finally, only associations between clinical and biomechanical measures were examined. The study therefore does not establish concurrent validity in a strict psychometric sense, nor does it define diagnostic thresholds for identifying impaired balance or fall risk. The findings should instead be interpreted as preliminary evidence regarding which clinical tests may best approximate specific laboratory-derived aspects of balance control in people with MS.

## Conclusion

Overall, although the small sample size precludes formal assessment of concurrent validity, the observed pattern tentatively suggests that the BBS may be more closely related to static postural steadiness, whereas the TUG may better reflect dynamic balance during walking. All clinical tests appeared to reflect balance ability during standing to a moderate or strong degree, whereas this was not equally the case for balance ability during walking. Except for the TUG, the clinical tests appeared to reflect the dynamic domain less strongly. Given the limited time typically available to therapists in routine clinical practice, these preliminary findings of this study suggest that the TUG may be a comparatively informative single clinical test for capturing aspects of both static and dynamic balance control in ambulatory people with mild-to-moderate MS-related disability.

## Supplementary Information

Below is the link to the electronic supplementary material.

Supplementary Material 1

## Data Availability

The dataset used and analyzed during the current study is available from the corresponding author upon reasonablerequest.
