# Supplementary material for: Distinct correlations between clinical and laboratory-based instrumented balance measures in individuals with multiple sclerosis: a cross-sectional study
Source: Sci Rep. 2026 Jun 23;16:28816. doi: 10.1038/s41598-026-58829-9 (PMC13578434; doi:10.1038/s41598-026-58829-9)
Supplement: Supplementary file 1 [file 41598_2026_58829_MOESM1_ESM.docx]

***Supplementary Table 1***

**Demographic and clinical characteristics of the participants**

| Participant | EDSS | Gender | Age range | Number of falls in the past 6 months |
| --- | --- | --- | --- | --- |
| 1 | 3.5 | f | 30-34 | 0 |
| 2 | 3.0 | f | 45-49 | 0 |
| 3 | 4.5 | m | 55-59 | 2 |
| 4 | 4.0 | f | 55-59 | 2 |
| 5 | 4.0 | f | 50-54 | 0 |
| 6 | 3.5 | f | 45-49 | 0 |
| 7 | 4.5 | f | 65-69 | 1 |
| 8 | 1.5 | f | 40-44 | 0 |
| 9 | 4.0 | m | 60-64 | 0 |
| 10 | 5.5 | f | 45-49 | 0 |
| 11 | 4.5 | m | 45-49 | 3 |
| 12 | 4.5 | m | 40-44 | 1 |
| 13 | 5.0 | m | 45-49 | 1 |
| 14 | 4.5 | f | 45-49 | 1 |
| 15 | 4.5 | f | 60-64 | 4 |
| 16 | 4.0 | m | 50-54 | 2 |
| 17 | 4.5 | m | 50-54 | 0 |
| 18 | 2.0 | m | 40-44 | 0 |
| 19 | 3.0 | f | 55-59 | 0 |
| 20 | 3.0 | f | 55-59 | 0 |
| 21 | 3.0 | f | 55-59 | 0 |
| 22 | 2.0 | m | 30-35 | 0 |
| 23 | 4.0 | f | 60-65 | 0 |
| 24 | 1.5 | f | 40-45 | 0 |
| 25 | 3.0 | f | 45-49 | 0 |
| 26 | 5.5 | f | 25-29 | 0 |
| 27 | 2.0 | f | 55-59 | 0 |
| 28 | 3.0 | f | 25-29 | 0 |
| 29 | 3.0 | f | 55-59 | 0 |
| 30 | 3.0 | f | 65-69 | 0 |

Participant characteristics. EDSS = Expanded Disability Status Scale

***Supplementary Figure 1***

Correlation matrix for all pairwise comparisons including r-values, bootstrap confidence intervals and uncorrected p-values. The r-values are color-coded (see scale), and the size of the background squares is scaled according to magnitude and direction. Associations are greyed out and displayed in italics if the bootstrap confidence interval included r = 0 or did not include |r| ≥ 0.5. BBS = Berg Balance Scale; CoP = Center of Pressure; CTSIB = Clinical Test of Sensory Interaction on Balance; DGI = Dynamic Gait Index; MoS = Margin of Stability; SSST = Six Spot Step Test; TUG = Timed Up and Go Test; WBAM = Whole-Body Angular Momentum; AP = anteroposterior; ML = mediolateral.

***Supplementary Table 2***

**Results of the clinical balance assessments and motion-analysis-derived measures**

| **Test** | **Mean** | **SD** |
| --- | --- | --- |
| MiniBest | 23.3 | 3.9 |
| DGI | 19.8 | 3.6 |
| TUG | 8.3 | 3.0 |
| SSST | 11.3 | 3.8 |
| BBS | 53.9 | 3.0 |
| CTSIB | 11.4 | 3.0 |
| CoP 95% ellipse (mm^2^) | 483.4 | 471.4 |
| MoS_ap | 20.1 | 60.7 |
| MoS_ml | 80.7 | 17.3 |
| WBAM sag. plane | 0.04 | 0.014 |
| WBAM front. plane | 0.06 | 0.017 |
| WBAM trans. plane | 0.02 | 0.004 |
